# Population Monitoring of the Red Junglefowl Based on Acoustic Signal Recognition Technology

**DOI:** 10.1002/ece3.71280

**Published:** 2025-04-16

**Authors:** Peipei Hao, Xingyi Jiang, Xiaodong Rao, Wei Liang, Yanyun Zhang

**Affiliations:** ^1^ Ministry of Education Key Laboratory for Biodiversity and Ecological Engineering, College of Life Sciences Beijing Normal University Beijing China; ^2^ School of Tropical Agriculture and Forestry 5 Hainan University Danzhou China; ^3^ Haikou Key Laboratory of Intelligent Forestry Haikou China; ^4^ Ministry of Education Key Laboratory for Ecology of Tropical Islands, Key Laboratory of Tropical Animal and Plant Ecology of Hainan Province College of Life Sciences, Hainan Normal University Haikou China

**Keywords:** automatic signal recognition, population size, red junglefowl, vocal individuality

## Abstract

Vocalisation is a crucial means of communication for birds and plays a key role in survival and reproductive success. Individual differences in songs have been used for identification in many animals, but few studies have integrated song individuality into wildlife population monitoring. The male red junglefowl *Gallus gallus jabouillei* is a tropical forest bird that primarily uses acoustic signals for conspecific communication. From July to August 2020, the calls of 34 pasture‐raised red junglefowl were recorded for individual identification based on vocalisations. Fieldwork was conducted from March to May 2021 in the Datian National Nature Reserve, Hainan, China, during which microphone arrays were deployed to record the calls of wild red junglefowl throughout their breeding season. Discriminant function analysis (DFA) was applied to identify pasture‐raised red junglefowl individuals, achieving a correct identification rate of 95.7%. Affinity propagation (AP) clustering was used to perform unsupervised clustering based on pairwise syllable similarities, resulting in 34 clusters corresponding to the actual number of individuals, with a correct syllable type recognition rate of 99.4%. Kaleidoscope software was used to extract the call during the breeding period of the wild population of red junglefowl; the precision rate was 80.38%, and the recall rate was 75.85%. Using AP clustering for vocalisation analysis, the estimated population in the core area was approximately 205 male individuals, with a manual verification accuracy of 82.5%. This result is slightly lower than the estimate of 234 individuals obtained using vocal count and random encounter methods. Our study demonstrated the potential of affinity propagation clustering techniques for estimating the population size of wild red junglefowl.

## Abstract 2

Vocalisations of red junglefowls ( *Gallus gallus jabouillei* ) are monotonous and stereotyped, with significant individual variation. Combination of passive acoustic monitoring and automatic vocal signal recognition technology can effectively track the vocal behaviour of red junglefowl. The use of affinity propagation clustering techniques allows for an effective estimation of population size of the wild red junglefowl.

## 1 Introduction

The global decline in biodiversity has created an urgent need for cost‐effective and scalable technologies to monitor wildlife populations (Cardinale 2012). In response, a range of non‐invasive technologies such as LiDAR, infrared camera traps, and DNA barcoding have recently been developed and introduced, expanding the toolkit for estimating wildlife density (Pimm et al. 2015). Among these, passive acoustic monitoring (PAM) has emerged as a particularly promising approach for studying vocalising species, offering new insights into biodiversity and population dynamics (Kotila et al. 2023; Serrurier et al. 2024).

PAM involves the deployment of autonomous recording devices in a study area to systematically capture acoustic data from animals and their environment according to a pre‐set schedule (Batist et al. 2024). This technology minimises human presence and associated disturbances while enabling data collection across broader spatial and temporal scales compared to traditional survey methods (Todd et al. 2022; Winiarska et al. 2024). With the progress in data storage and artificial intelligence (AI), PAM has become a powerful tool for wildlife research, particularly for species that are hard to observe directly (Wrege et al. 2017; Wood et al. 2019). By analysing acoustic recordings, researchers can infer the presence or absence of target species, estimate the number of vocalising individuals, and derive population density metrics (Clink et al. 2021; Manzano‐Rubio et al. 2022).

However, the widespread application of PAM is hindered by several challenges. Automated recorders can be expensive, and analyzing and processing large acoustic datasets require a significant amount of time and expertise (Pérez‐Granados and Traba 2021). Although recent developments in low‐cost recording technology and computing power for audio interpretation may address some of these issues (Marques et al. 2012), a major drawback persists: the lack of reliable techniques for accurately distinguishing between calling individuals (Darras et al. 2018). For example, assessing bird population densities using spatially explicit capture–recapture models—which rely on acoustic localisation or assigning calls to specific individuals based on acoustic characteristics—requires significant analyst effort (Efford et al. 2009; Harmsen et al. 2020). Hence, in terms of monitoring populations using vocal individuality, most existing studies have focused on data from a single season, limiting their ability to reflect long‐term population dynamics (Weinstein et al. 2022). While large–scale population monitoring struggles with individual distinction, machine learning holds the potential to overcome this limitation and greatly enhance analysis efficiency (Ferreira et al. 2020).

In machine learning, classification can be performed by supervised learning when the dataset is labelled (e.g., known species or individual identities) (Clink et al. 2017). For example, supervised learning algorithms can classify calls by species or individual identity when the identities of vocalising birds are known (Bocaccio et al. 2023). However, long‐term automatically collected acoustic data usually lack individual identity information. In such cases, unsupervised clustering algorithms can be used to infer individuals from unlabelled data (Hammerschmidt and Fischer 2019). Common unsupervised clustering methods include K‐means clustering, DBSCAN (density‐based spatial clustering of applications with noise) and affinity propagation (AP) clustering. Among these methods, AP clustering has been shown to outperform other unsupervised algorithms in various applications, including unsupervised classification of images, videos, and recordings (Dueck and Frey 2007; Clink et al. 2021). The significant advantage of AP clustering is that it does not require presetting the number of clusters and can transform acoustic features into similarity matrices, thus providing accurate individual identification for species population monitoring (Fischer et al. 2018; Yan et al. 2021). This capability makes AP clustering a powerful tool for biodiversity monitoring and conservation efforts, particularly for elusive or endangered species.

The red junglefowls *Gallus gallus jabouillei* inhabit cryptic environments and are highly vigilant, making direct observation difficult (Rao et al. 2023). During the breeding season, males engage in elaborate displays, including fluffing their feathers, spreading their wings, and making distinctive calls to attract females (Akrim et al. 2016). Among them, acoustic signals play a crucial role in their social interactions, with males emitting a territorial call of “ge‐ga‐ga‐ge” during the breeding season (Hao and Zhang 2020). Compared with those of other species, the vocal structure of the red junglefowl is relatively simple, making their calls easy to analyse and ideal for individual identification through vocalisation. Between 2021 and 2022, large‐scale vegetation burning and clearing by the Datian National Nature Reserve, Hainan Province, China, aimed at improving the habitat for Hainan Eld's deer *Rucervus eldii* , also indirectly affected the local red junglefowl population. In the core area of the reserve, the red junglefowl population has become a relatively closed group, with limited immigration or emigration, providing an ideal setting for studying population dynamics through PAM.

This study provides a comprehensive analysis of the vocal structure of the male red junglefowl, aiming to address the following questions: (1) Does the vocalisation of the red junglefowl exhibit individuality? If so, (2) can individual vocal characteristics in combination with the PAM provide reliable estimates of the red junglefowl population size?

## 2 Materials and Methods

### 2.1 Acoustic Data Collection

**FIGURE 1:**
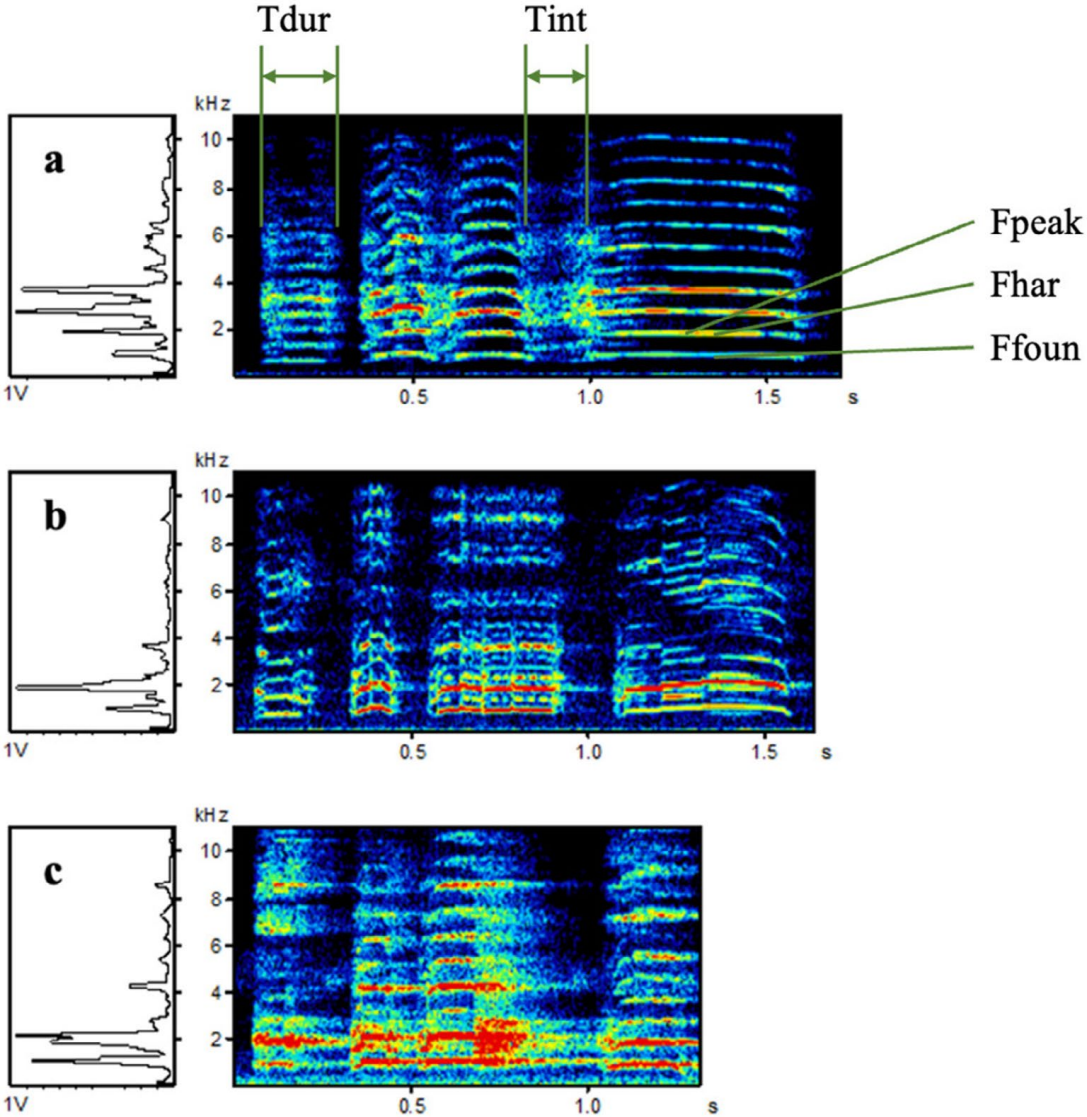
Spectrograms of the calls of male red junglefowl. (a) 9‐month‐old pasture‐raised red junglefowl (GS), (b) 18‐month pasture‐raised red junglefowl (GA) and (c) wild red junglefowl. Each call measured the duration of each note (Tdur1, Tdur2, Tdur3, and Tdur4), the interval length of the notes (Tint1, Tint2, and Tint3), the peak frequency of each notes (Fpeak1, Fpeak2, Fpeak3, and Fpeak4), the fundamental frequency (Ffoun1, Ffoun2, Ffoun3, and Ffoun4) and first harmonics (Fhar1, Fhar2, Fhar3, and Fhar4) as well as the duration of the syllable (Total).

The study was conducted at two locations: the chicken farm (19°37′ 24" N, 110°12' 56" E) and the Datian National Nature Reserve (19°05′‐17′ N, 108°71′‐88′ E). The chicken farm is located in Xinzhu town, Ding'an County, Hainan Province, and covers an area of 0.3 km^2^. In July 2021, we collected calls from 20 subadults (9 months old; *Gallus* subadults, hereafter GS) and 14 adults (18 months old; *Gallus* adults, hereafter GA) of pasture‐raised red junglefowls for recording (Figure 1). The individuals were artificially bred and had undergone approximately 7–8 generations of free‐range breeding. They were recorded over 10 days to assess the feasibility of using male red junglefowl calls for long‐term individual identification. Recordings were conducted from 06:00–10:00 a.m., during the red junglefowl's peak vocalisation period, to ensure clear spectrograms. Each bird individual was placed in a separate chicken coop, at least 15 m apart, to prevent vocal interference. Recordings were made using a TASCAM DR‐100MKIII recorder (TASCAM, Japan) and a Sennheiser MKH416P48 directional microphone (Sennheiser, Germany). The parameters were set as follows: sampling rate, 22,050 Hz; sampling accuracy, 16 bits; and file format, WAV.

**FIGURE 2:**
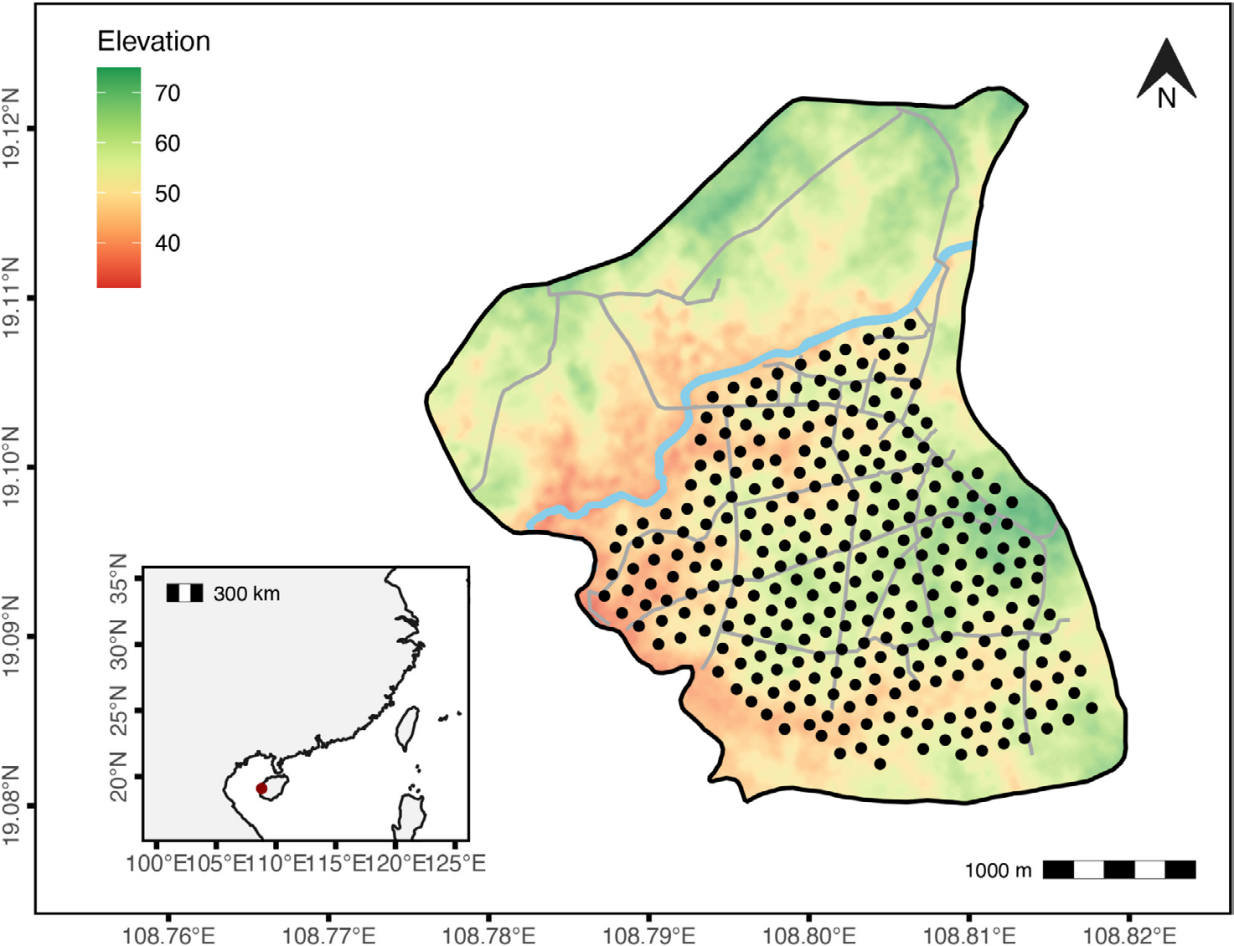
Study area. Each dot represents a recording location. The light blue line represents the river, and the grey line represents the road.

The Datian National Nature Reserve, located in Dongfang City, Hainan Province, primarily conserves the habitat for Hainan Eld's deer (Figure 2). The reserve is relatively flat, and it covers an area of 1310 ha (Rao et al. 2023). From March to May 2021, we established a PAM network in the reserve to conduct an acoustic monitoring during the peak vocalisation periods of red junglefowl. The 55 automatic recorders (Wildlife Acoustics, USA, Song Meter SM4) were mounted on trees at heights of 1.5–2 m. The duty cycle was set for one hour before and after sunrise and one hour before and after sunset. Recorders were spaced approximately 150 m apart within each device's effective recording range to ensure coverage of all individuals (Pérez‐Granados and Schuchmann 2020; Pérez‐Granados and Schuchmann 2023). After recording for 5 days, the microphone array was relocated to cover new areas until the core reserve area was completely surveyed. In total, the array was moved six times, covering 292 points and approximately 705 ha. The recording parameters of the units were configured as follows: sample rate, 22,050; gain, 16 dB; maximum length, 1 h; and file format, WAV.

### 2.2 Line Transect Survey of the Red Junglefowl Population

From March to April 2022, during the morning peak of vocalisation of red junglefowl (5:30–9:00), we conducted transect surveys along trails and firebreaks in the reserve at a consistent pace. The walking speed along the transects was 1–2 km/h, and we recorded the number of calling males, the composition of the breeding groups, and the sex ratio along the way. Each transect was surveyed every month for 3 consecutive days, and the surveys were conducted on sunny days to avoid the impact of weather on vocalisations. The transects were 4.18 km, 5.29 km, and 5.93 km in length, with a survey zone extending 150 m on each side, covering a survey area of approximately 4.62 km^2^. During the 3‐day survey of each transect, the highest number of male birds was taken as the number for that transect. The data are expressed as the means ± standard error (SE). The population size was calculated using the following formula (Yuan 2009):

$$\text{Number of males}=\frac{\text{Number of males recorded in transect surveys}\times \text{Size of the core area}}{\text{Survey area}.}$$

### 2.3 Wild Red Junglefowl Calls Extraction

We used Avisoft‐SASLab Pro 5.2 software (Avisoft Bioacoustics, Germany) to convert the recorded files into spectrograms for visual analysis. Ten recordings were randomly selected from different recorders, from which 50 clear spectrograms of wild red junglefowl calls were selected to measure the acoustic parameters and determine the time‐frequency parameters of automatic recognition (Table 1). The spectrogram parameters included a fast Fourier transform (FFT), a Hamming window, a frame size of 100%, an overlap of 75%, and a temporal resolution of 2.9025 ms.

**TABLE 1:** Acoustic parameters of red junglefowl calls (mean ± SE).

Duration (s)	Minimum frequency (Hz)	Maximum frequency (Hz)	Peak frequency (Hz)
1.36 ± 0.01 (1.09–1.50)	805.20 ± 14.85 (600–1110)	3072.60 ± 127.83 (1550–5340)	1251.20 ± 48.60 (940–2320)

*Note:* The contents of the parentheses indicate the minimum and maximum values.

Kaleidoscope Pro 5.1.9 (Wildlife Acoustics, USA) was used to extract red junglefowl calls in the left channel of the recordings. Kaleidoscope is an automatic signal recognition software that examines recordings for signals of interest on the basis of the time–frequency parameters of the input. Since the high frequency signal attenuates as the distance increases, the highest frequency of the call varies greatly, so the peak frequency of the call can be used as a reliable indicator to accurately characterise the call signal. According to the results of the call measurements (Table 1) and the pre‐tests, we finally selected signal parameters with minimum and maximum frequency range (850–1650 Hz), call lengths (1.0–2.0 s, respectively), and maximum syllable intervals of 0.2 s as candidates. The model was established by extracting vocalisation features using a discrete cosine transform (DCT) and optimising the red junglefowl call model via human‐assisted machine learning. Each cluster automatically created by Kaleidoscope was manually labelled ‘Gallus’ or ‘Other’ depending on whether target vocalisations appeared within the top 50 candidates for each cluster (Pérez‐Granados and Schuchmann 2020). The clusters labelled ‘Others’ were excluded from subsequent analysis and were not examined. Visual and/or acoustic examinations were conducted by the same professional (Peipei Hao) on each candidate vocalisation in the ‘Gallus’ cluster to exclude nontarget vocalisations.

Kaleidoscope does not directly report scores for the automatic identification of sound signals but instead uses a clustering method to report the distances between candidate calls. Therefore, the reciprocal of the distance to the cluster centre was used as an alternative to the score threshold, and the score threshold was normalised using range normalisation (Knight et al. 2017). To evaluate the clustering analysis functionality of Kaleidoscope, the precision and recall rates identified by the software were measured. The precision rate was calculated as the proportion of target calls (true positives, TP) among all automatically identified candidate sounds, expressed as TP/(TP + FP), where FP represents false positives (incorrect extractions). That is, the number of true positives (correct extractions) is divided by the total number of candidate calls in the ‘Gallus’ cluster. The recall rate represents the proportion of target calls (true positives) automatically identified by the software out of the total vocalisations present in the recordings, calculated as TP/(TP + FN), where FN is the number of false negatives (the number of red junglefowl vocalisations missed by the detector). This is the number of true positives (correct extractions) divided by the total number of vocalisations (Hao and Zhang 2020). The testing set consisted of approximately 100 GB of recording data, with time‐frequency parameters adjusted to maximise the extraction of red junglefowl calls. Experts manually screened and labelled each detection to identify true positives, which were used as the total number of vocalisations. The number of red junglefowl calls accurately extracted by the previously established model was used as the true positives in the test set.

### 2.4 Sound Measurements and Individual Identification

#### 2.4.1 Pasture‐Raised Red Junglefowl

We selected 1911 calls from the GS and 646 calls from the GA for analysis. Each spectrogram measured three temporal variables, including the duration of each note (Tdur1, Tdur2, Tdur3, and Tdur4), the interval length of the notes (Tint1, Tint2, and Tint3), and the duration of the syllable (Ttotal). Additionally, three structural variables were measured, including the peak frequency of each note (Fpeak1, Fpeak2, Fpeak3, and Fpeak4), the fundamental frequency (Ffoun1, Ffoun2, Ffoun3, and Ffoun4), and the primary harmonic (Fhar1, Fhar2, Fhar3, and Fhar4). We measured 32–197 calls per individual (mean ± SE = 75.21 ± 7.39) and used the mean for subsequent analysis. As there were no significant differences in the acoustic parameters between the GA and GS, all vocalisations were analysed together (Hao and Zhang 2020). We calculated the coefficient of variation (CV) for each variable and then compared the differences within individuals and among individuals. The ratio of the among‐ to within‐individual coefficient of variation (CVa/CVw) was calculated as the potential individual coding (PIC) to assess each variable's effectiveness for individual identification (Chen et al. 2020). If the PIC value is greater than 1, the among‐individual difference of this variable is greater than the within‐individual difference, making it suitable for individual identification. If the PIC value is less than 1, the variable is not subsequently analysed. From the total pool of recordings, 10 recordings per bird individual were randomly selected to serve as the training set for establishing the discriminant function analysis (DFA), while the remaining 2217 recordings were used as the testing set.

We selected 5 clear calls for each individual and saved them as son files. Using Avisoft‐SASLab Pro software, background noise below 400 Hz was removed, and the frequency deviation was set to 50 Hz. The ‘template cross‐correlation on short files’ function was then applied to perform spectrogram cross‐correlation analysis (SPCC), calculating similarity values between pairs of syllables (Deng et al. 2019). The Hopkins statistic, ranging from 0 to 1, was used to assess the clustering tendency of similarity values, where values closer to 1 indicate stronger clustering. The AP clustering algorithm was used for unsupervised clustering of the similarity values between syllables. AP clustering uses a similarity matrix to represent the similarity between data points and determines the cluster centres by passing messages between data points (Brusco et al. 2019). During the clustering process, two types of messages are passed between nodes: attraction r (i, k) and responsibility a (i, k). Attraction r (i, k) indicates how well point k serves as the cluster centre for point i, whereas responsibility a (i, k) indicates the appropriateness of point k serving as the cluster centre for point i, considering that other points might also serve as cluster centres. Through an iterative process, the attraction and responsibility values of each point are continuously updated until m high‐quality exemplars (similar to centroids) are generated, and the remaining data points are assigned to the corresponding clusters (Clink et al. 2021). The classification accuracy was calculated based on known vocalisation labels for pasture‐raised red junglefowl individuals. The similarity between vocalisations of the same and different individuals was tested using the K–S test.

#### 2.4.2 Acoustic Analysis of Wild Red Junglefowl Vocalisations

Using Kaleidoscope software's automatic signal recognition results, vocalisation signals recorded by each recorder during peak vocalisation periods (1 h before and after sunrise) were examined visually and/or aurally. Red junglefowl individuals often emit a series of repetitive calls at the peak of the calling period, and the calls show significant individual variation, which makes it easy to distinguish the number of males around the recorder. However, since wild red junglefowl are mobile and the distance between recorders is smaller than the species' detection range, vocalisations from the same individual may be captured by multiple recorders, leading to pseudo‐replication. To address this issue and ensure the accuracy of the algorithm, based on preliminary differentiation, 3 clear calls were selected from each individual, and a total of 1002 calls were selected from 292 recorders. Avisoft‐SASLab Pro software was used to perform spectrogram cross‐correlation analysis, quantify differences between individual calls, and calculate similarity scores for each pair of calls. The Hopkins statistic was used to test the spatial clustering of similarity values. Moreover, AP clustering was used to cluster the similarity values between pairs of vocalisations based on the similarity matrix. Then, 200 red junglefowl calls were randomly selected from a total of 1002 calls. The accuracy of the clustering results was tested using call pair similarity values and the home range size (95% of the red junglefowl home ranges are approximately 11 ha; Hao and Zhang, unpublished data) of the red junglefowl. A call was considered correctly categorised if it exhibited high similarity to other calls in the same group and the recording locations were within the home range of the red junglefowl. Otherwise, the call was classified as misclassified. All the statistical analyses were conducted in R 4.3.1, with the significance level set at *p* < 0.05.

## 3 Results

### 3.1 Acoustic Identification of the Pasture‐Raised Red Junglefowl

Through the analysis of the vocalisations of 34 pasture‐raised red junglefowls, the intraindividual coefficient of variation (CV) for GA vocalisations ranged from 0.03–0.44, and the interindividual CV ranged from 0.08–0.85, with PIC values ranging from 0.83–12.07. For GS, the intraindividual CV ranged from 0.05–1.04, and the interindividual CV ranged from 0.08–1.25, with PIC values ranging from 0.34–4.71 (Table 2). The vocalisations of the GA and GS were combined for individual recognition, and 16 of the 20 time and frequency parameters measured were considered suitable for individual recognition (PIC > 1). The PIC values for the peak frequencies of each note (Fpeak1, Fpeak2, Fpeak3, and Fpeak4) were less than or very close to 1, indicating that within‐individual differences in these variables are less than or equal to among‐individual differences, so these four variables were not used in subsequent analyses.

**TABLE 2:** The coefficients of variation within (CVw), among (CVa) call variations and their ratio in syllable.

Variables	GS			GA		
	CV among	CV within	PIC	CV among	CV within	PIC
Tdur1	0.21	0.11	**1.96**	0.18	0.07	**2.50**
Tdur2	0.23	0.08	**2.84**	0.64	0.05	**12.07**
Tdur3	0.29	0.06	**4.71**	0.40	0.06	**6.86**
Tdur4	0.65	0.16	**4.17**	0.31	0.12	**2.58**
Total	0.13	0.05	**2.55**	0.08	0.03	**2.62**
Tint1	0.28	0.15	**1.80**	0.27	0.11	**2.45**
Tint2	1.25	1.04	**1.21**	0.75	0.44	**1.72**
Tint3	0.96	0.81	**1.18**	0.85	0.27	**3.21**
Fpeak1	0.13	0.37	0.34	0.25	0.29	0.86
Fpeak2	0.12	0.22	0.57	0.21	0.26	0.83
Fpeak3	0.13	0.29	0.44	0.21	0.22	0.93
Fpeak4	0.18	0.26	0.69	0.18	0.18	1.03
Ffoun1	0.12	0.06	**2.16**	0.11	0.06	**2.02**
Ffoun2	0.08	0.07	**1.16**	0.26	0.05	**5.03**
Ffoun3	0.10	0.05	**2.01**	0.08	0.05	**1.43**
Ffoun4	0.14	0.06	**2.40**	0.37	0.05	**7.72**
Fhar1	0.10	0.06	**1.56**	0.13	0.05	**2.39**
Fhar2	0.09	0.06	**1.56**	0.10	0.05	**1.95**
Fhar3	0.08	0.05	**1.57**	0.16	0.05	**3.14**
Fhar4	0.11	0.05	**2.13**	0.08	0.04	**2.16**

*Note:* Values used for individual identification are bolded.

**FIGURE 3:**
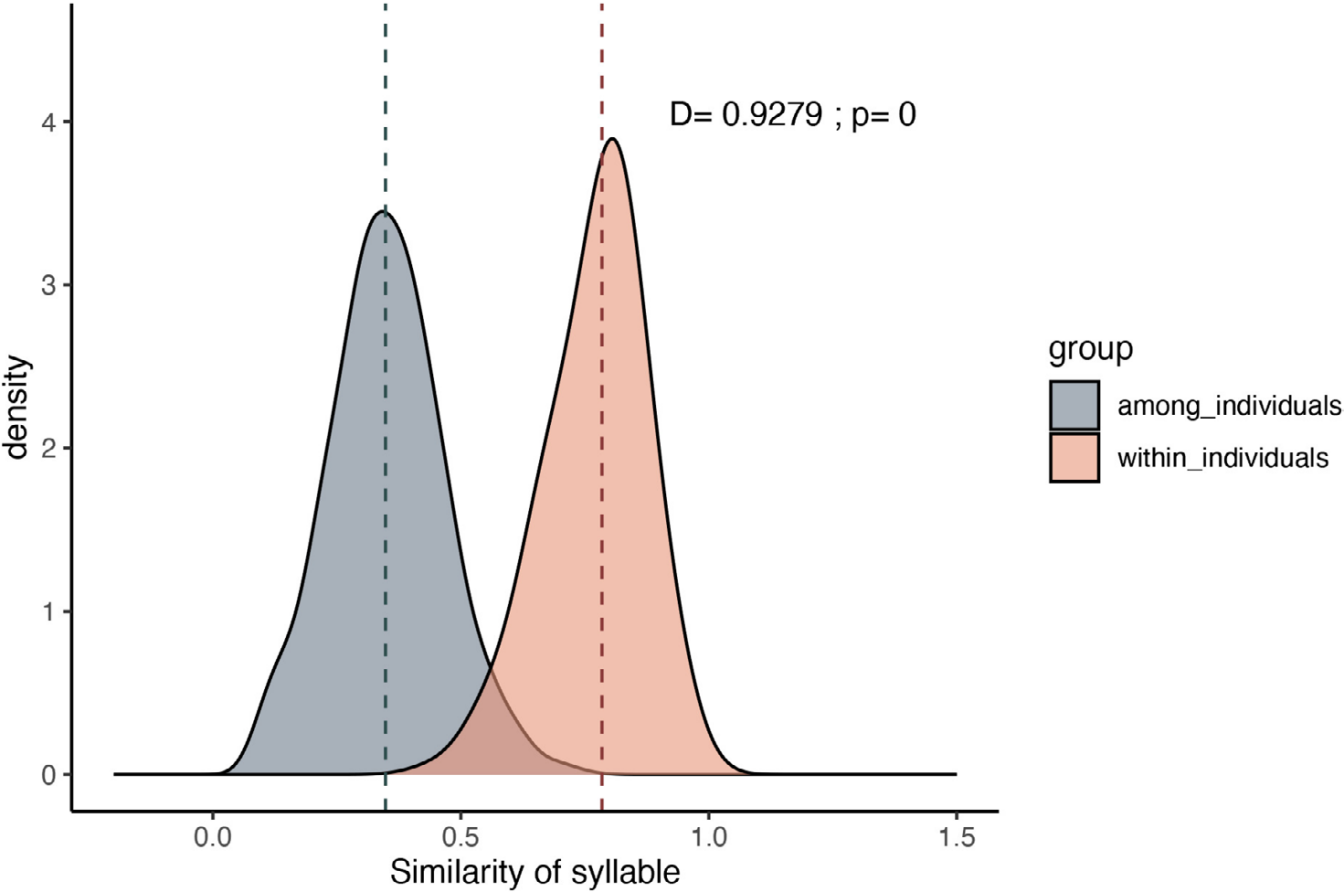
Density map of correlation coefficients of pair calls from the same male and different males. The dotted line indicates the median value.

DFA was used to identify individuals in 2217 red junglefowl calls, achieving a correct identification rate of 95.70%, indicating that 2121 calls were accurately assigned to their respective individuals. On the basis of the spectrogram similarity results, the similarity between syllable pairs from the same individual (mean ± SD = 0.71 ± 0.10) was significantly greater than that between different individuals (0.35 ± 0.12) (K‐S test: D = 0.93, *p* < 0.01) (Figure 3). The Hopkins statistic (0.88) indicated strong clustering in the data (greater than the 0.5 threshold). Using AP clustering on the similarity values between pairs of syllables, the clustering results identified 34 individuals, which is consistent with the actual number. Among the 170 red junglefowl vocalisations, 169 were correctly classified, with an individual identification accuracy rate of 99.41%.

### 3.2 Acoustic Identification of the Wild Red Junglefowl

**FIGURE 4:**
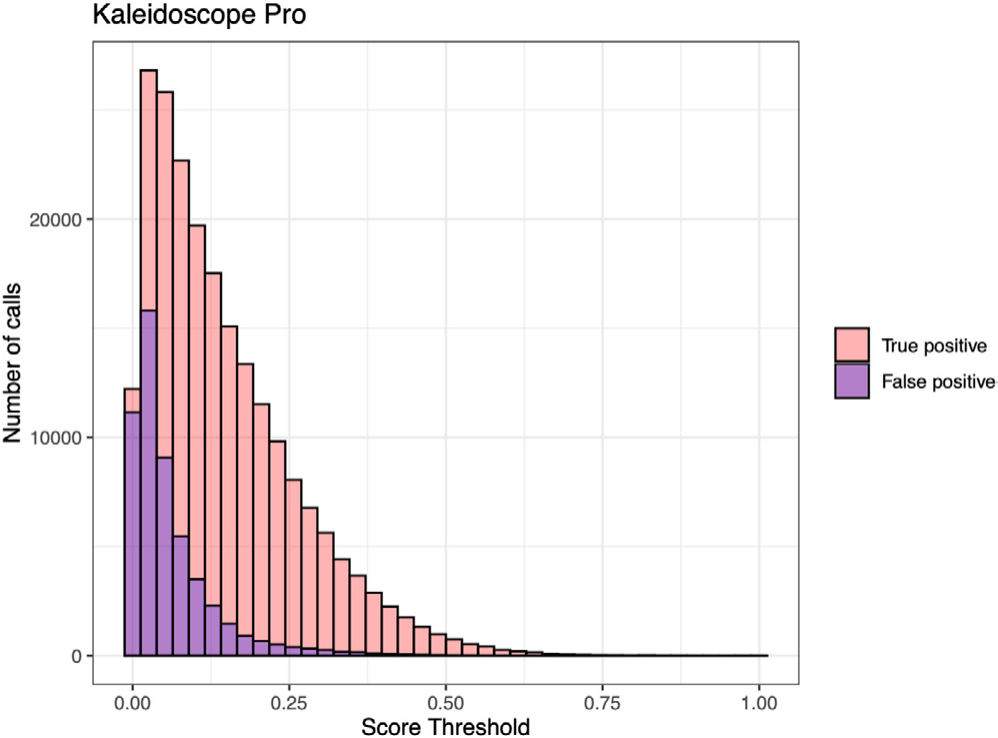
Distribution of true positive and false‐positive recogniser calls relative to the score.

After optimising the time‐frequency parameters, machine‐assisted recognition processed approximately 7 TB of breeding season recordings, extracting 267,110 vocalisations with 214,691 correctly identified, achieving a precision rate of 80.38%. As the score threshold increased, both the true and false‐positive rates significantly decreased (Figure 4). From the 100 GB test set, a total of 10,864 red junglefowl vocalisations were detected, with 8240 correctly identified by the previously established model, resulting in a recall rate of 75.85%. A Hopkins statistic of 0.91 (> 0.50) indicated a strong clustering tendency within the dataset, suggesting a robust cluster structure. Using AP clustering on the correlation values between pairs of calls, the clustering results identified 205 male individuals. Among the 1002 junglefowl vocalisations, 200 were randomly selected for manual verification (using both audio and spectrograms), and 165 were found to be correctly classified, achieving a classification accuracy of 82.5%.

In March–April 2022, traditional vocalisation counting methods were used to assess the breeding population of the red junglefowl. The March survey estimated 79 males, whereas the April survey indicated an increase to 153 males. Therefore, the number of males in April was taken as the estimate for the number of males in the sample transect area. On this basis, the estimated number of male red junglefowls in the core area of the reserve was approximately 234 birds. The transect surveys recorded a total of 39 breeding groups comprising 94 individuals, including 46 females and 48 males, with an average group size of 2.41 ± 0.11 (range: 2–5) individuals per group.

## 4 Discussion

Our study revealed that calls of the male red junglefowl exhibit individuality, with greater among‐individual variation than within‐individual variation. Acoustic monitoring has the potential to assess the size of red junglefowl populations based on the individual characteristics of vocalisations and traditional transect surveys monitoring the wild population. This provides a case study for individual identification in avian population research, contributing to a deeper understanding of the vocalisations and breeding behaviour of red junglefowls.

### 4.1 Acoustic Identification of Pasture‐Raised Red Junglefowl

The analysis of pasture‐raised red junglefowl calls revealed 16 temporal and frequency parameters with high potential for individual identification (PIC values > 1). The individuality of vocalisations has been widely studied in non‐passerine birds, especially in social or nocturnal species (Osiejuk et al. 2019; Klenova et al. 2020; Oñate‐Casado et al. 2023), but research on vocal individuality in Galliformes is relatively limited (Hale et al. 2014; Hambálková et al. 2021). Previous studies have shown that the temporal parameters of the Japanese Quail *Coturnix japonica* vocalisations may carry information that can be used by females during mate selection, and these parameters have strong potential for individual identification (Sezer and Tekelioglu 2010). The hissing calls of the Black Grouse *Tetrao tetrix* during mating display individuality, helping females select mates and aiding males in recognising and evaluating potential competitors (Hambálková et al. 2021). The low‐frequency vocalisations of male Western Capercaillie *Tetrao urogallus* show significant individual differences, which aid in transmitting information over long distances and conveying details about male quality (Hart et al. 2020). In this study, DFA and spectrogram correlation methods, combined with AP clustering, achieved a correct identification rate of over 95%. Furthermore, the pasture‐raised red junglefowl were more closely related to each other and had a higher degree of vocal similarity. This high accuracy highlights the potential of acoustic monitoring for individual identification and provides a solid foundation for future population monitoring and individual identification of wild red junglefowl.

### 4.2 Automatic Signal Recognition of Wild Red Junglefowl Vocalisations

PAM offers significant advantages for large‐scale and long‐term monitoring of avian populations, enabling simultaneous sampling across extensive temporal and spatial scales (Sugai et al. 2019). However, the effectiveness of PAM depends on the accuracy of automated signal recognition software (Stowell et al. 2019). In this study, the precision and recall rates for automatic detection of wild red junglefowl calls were 80.38% and 75.85%, respectively, consistent with previous studies using similar software (Pérez‐Granados and Schuchmann 2021; Manzano‐Rubio et al. 2022). Using the software Kaleidoscope for song extraction of the Striped Cuckoo *Tapeta naevia*, the recall rate was 78%–84%, but the precision rate of the recognizer was very low, at 5%–14% (Pérez‐Granados and Schuchmann 2023). Although the accuracy of the recognizer did not impact our results as each candidate sound in the ‘Gallus’ cluster was manually verified, the labor—intensive nature of this verification process may pose challenges for future applications (Pérez‐Granados and Schuchmann 2022). In the ‘Gallus’ cluster, most of the misclassified sounds were calls of birds that sing at the same frequency as the red junglefowl, such as the Jungle Nightjar *Caprimulgus indicus* , the Chinese Francolin *Francolinus pintadeanus* , and the Collared Scops Owl *Otus lettia* . Employing machine learning to develop advanced classifiers may increase accuracy, ultimately minimising the occurrence of misclassified sounds (LeBien et al. 2020). For example, convolutional neural networks (CNNs) have outperformed traditional recognizers like Raven Pro and Kaleidoscope in species with complex vocal repertoires (Knight et al. 2017). A recent study showed that CNN model trained on a large dataset of bird vocalisations can significantly improve the accuracy of individual recognition of Yellow Cardinal *Gubernatrix cristata* (Bocaccio et al. 2023). Integrating such techniques into PAM workflows could enhance the accuracy and efficiency of population monitoring.

### 4.3 Acoustic Identification of Individual Wild Red Junglefowl

PAM has emerged as a powerful tool for estimating population size and density in avian species, particularly for those with distinctive vocalisations (Efford et al. 2009; Marques et al. 2012; Measey et al. 2017). After collecting calls using automated recorders, information on the number of individuals vocalising around the recorder can be further investigated to expand monitoring beyond detecting the presence of the species (Manzano‐Rubio et al. 2022). In a study monitoring the vocal activity of male Western Capercaillies *Tetrao urogallus* at a lek, a significant positive correlation was found between the number of male individuals and their vocalisations (Abrahams 2019). Monitoring of the Cory's Shearwater *Calonectris diomedea* and the Common Bee Eater *Merops apiaster* revealed a linear positive correlation between the acoustic activity rates and the population abundance and acoustic activity rates of Dupont's Lark *Chersophilus duponti* , which exhibited a logarithmic correlation with the population abundance (Oppel et al. 2014; Pérez‐Granados et al. 2019). A population density assessment of the Bell Miner *Manorina melanophrys* involving a combination of visual surveys with acoustic monitoring revealed that acoustic monitoring detected more individuals and greatly reduced the need for researcher expertise (Lambert and McDonald 2014). These findings highlight the potential of PAM for estimating population size in species with distinct vocal behaviours. During the breeding season, red junglefowls emit a series of monotonous, loud territorial calls, and these calls exhibit strong individuality. The use of automated recorders and AP clustering allowed us to estimate the population size of wild red junglefowl, identifying 205 male individuals with an identification accuracy of 82.5% after manual validation. The AP clustering algorithm uses a similarity matrix as input, making it widely applicable to various types of datasets, especially in bioacoustics and PAM, where multivariate datasets are common (Dueck and Frey 2007). In distinguishing the vocal data of the female Northern Grey Gibbon *Hylobates funereus* , AP clustering outperformed K‐medoids and Gaussian mixture model‐based clustering in monitoring monotonous, stereotyped vocal animals (Clink et al. 2021). Our findings showed that unsupervised clustering based on AP clustering can be a valuable supplement to population density estimation and is essential for future effective monitoring of population status and trends of endangered species.

When comparing population size estimates obtained through call counting and random encounter methods, the results revealed that line‐transect survey estimates were 12% greater than individual identification‐based estimates. This discrepancy may arise from two key factors: (1) repeated counts of the same individuals during transect surveys and (2) microphone arrays were moved six times during the survey period, with recordings lasting from early March to the end of April. In the early breeding season, some males had low or even essentially no calling activity, which may have led to an underestimation of population size by acoustic monitoring. Similar findings have been reported in other studies comparing acoustic monitoring with manual counts (Pérez‐Granados and Traba 2021). For example, species recall rates using point‐count methods were higher than those obtained through acoustic monitoring because of differences in species detection radii (Hutto and Stutzman 2009). Pairing manual counts with automatic recorder‐based sound sampling models revealed that recall rates on automatic recorders were slightly lower than the actual values for most species (van Wilgenburg et al. 2017). Although the study has provided initial comparisons between different population size estimation methods, paired sampling (simultaneous field surveys and recordings) over the same time period is lacking. Further validation through paired sampling is required thereafter to confirm the accuracy and applicability of the methodology in the natural environment.

Moreover, combining acoustic data with spatially explicit capture‐recapture models (SECRs) has shown promise in improving population density assessments (Efford et al. 2009; Harmsen et al. 2020; Strampelli et al. 2022). SECRs account for spatial heterogeneity in detection probabilities, providing more accurate estimates than traditional methods (Morris et al. 2022; van Heezik et al. 2023). In population density assessments of taxa such as chimpanzees *Pan troglodytes schweinfurthii* , bowhead whales *Balaena mysticetus* , and little spotted kiwi *Apteryx owenii* , the SECR model generally outperforms traditional capture‐recapture models in terms of statistical performance (Moore and Vigilant 2014; Kim et al. 2018; Juodakis et al. 2021). Combining traditional survey methods with a vocal recognition‐based SECR model to investigate the population size of the Critically Endangered cao vit gibbon *Nomascus nasutus* revealed that traditional survey methods overestimated the population size by nearly 38% due to repeated counts of the same individual (Wearn et al. 2024). In the future, SECRs can be applied to acoustic data of red junglefowl to further refine population estimates and account for variation in detection probabilities across habitats.

## 5 Conclusion

Given the global decline in biodiversity, effectively monitoring population dynamics is critical for the conservation of entire taxa. This study confirms that the vocalisations of the male red junglefowl exhibit individual specificity, characterised by significant interindividual differences and intraindividual stability, making them suitable for individual identification. Combining passive acoustic monitoring with automatic signal recognition software was effective in tracking the vocal behaviour of the red junglefowl. Additionally, integrating unsupervised clustering methods enabled preliminary estimation of the population size of the red junglefowl, with results closely aligning with those of traditional transect surveys. However, it is important to note that this method was first validated on pasture‐raised birds and then applied to wild populations, and its performance in real‐world conditions requires further validation. Future studies should incorporate paired sampling (simultaneous recordings and field surveys) to confirm the method's accuracy and applicability in natural settings. This approach not only provides a novel application of individual identification in avian population studies but also offers new perspectives and methodologies for assessing the population size of endangered pheasant species.

## Author Contributions

**Peipei Hao:** formal analysis (equal), investigation (equal), visualization (equal), writing – original draft (equal). **Xingyi Jiang:** investigation (equal). **Xiaodong Rao:** investigation (equal). **Wei Liang:** writing – review and editing (equal). **Yanyun Zhang:** conceptualization (equal), supervision (equal), writing – review and editing (equal).

## Conflicts of Interest

The authors declare no conflicts of interest.

## Data Availability

The data and the codes created in the research are available at https://github.com/peipeiiiii/Population‐monitoring.
